# Upregulated *FKBP1A* Suppresses Glioblastoma Cell Growth via Apoptosis Pathway

**DOI:** 10.3390/ijms232314935

**Published:** 2022-11-29

**Authors:** Shaoyi Cai, Zhiyou Chen, Heng Tang, Siyan Meng, Liang Tao, Qin Wang

**Affiliations:** Department of Pharmacology, Zhongshan School of Medicine, Sun Yat-Sen University, Guangzhou 510080, China

**Keywords:** *FKBP1A*, GBM, bulk RNA-seq, scRNA-seq, pathway enrichment, WGCNA

## Abstract

Glioblastoma (GBM), the most deadly primary brain tumor, presents a major medical difficulty. The need for better therapeutic targets in GBM is therefore urgent. A growing body of evidence suggests that the gene *FKBP1A* plays an important role in tumor progression and may be therapeutically useful. However, the role of *FKBP1A* in glioblastoma and the underlying biologic mechanism remain unclear. The purpose of this study was to identify the role of *FKBP1A* in GBM and its molecular mechanism. We demonstrated that *FKBP1A* was the hub gene in GBM via a weighted correlation network analysis (WGCNA) and differentially expressed genes (DEGs) analysis based on the bulk RNA-seq data from TCGA and GTEx. Afterwards, we proved that the upregulated *FKBP1A* protein could promote GBM cell death by CCK-8 assays in U87MG and t98g GBM cell lines. We further demonstrated two key pathways of *FKBP1A* in GBM by bioinformatics methods: ‘Apoptosis’ and ‘mTOR signaling pathway’. Subsequently, the key pathways were verified by flow cytometry and Western blot. We identified that upregulated *FKBP1A* could inhibit GBM growth via the apoptosis pathway. Together, these findings may contribute to future GBM treatment.

## 1. Introduction

Glioblastoma (GBM), the most common and deadly primary brain tumor, is deemed to derive from glial cells and has a highly malignant phenotype with rapid disease progression and a median survival of less than 1.5 years [1,2]. Currently, there is no optimized treatment for GBM, and the comprehensive treatment options of routine surgical resection combined with subsequent chemotherapy and temozolomide adjuvant therapy exhibit limited effectiveness [3,4]. Therefore, new targets and strategies for GBM treatments are urgently needed.

The *FKBP1A* protein (FKBP prolyl isomerase 1A, also known as FKBP12) was initially defined as an immunophilin for binding with Rapamycin and FK506 [5]. Recently, accumulating evidence suggested that *FKBP1A* plays a significant role in the development of tumors and is a possible therapeutic target. The *FKBP1A*/Rapamycin complex acts on the mammalian target of Rapamycin (mTOR) by repressing its serine/threonine phosphatase activity to achieve the effect of cell cycle arrest and cell growth repression. Unlike the tumor-suppressing mechanism produced by Rapamycin binding, individual *FKBP1A* overexpression inhibits tumors through a mechanism that is at least partially related to cell invasion pathways [6]. In addition, as *FKBP1A* degrades MDM2, it makes cells susceptible to chemotherapy-induced apoptosis; in breast cancer patients with high MDM2 expression, *FKBP1A* elevated sensitivity to anthracycline chemotherapeutic agents [7,8]. *FKBP1A* is essential for the effective and durable inhibition of mTORC1 and proliferation in human glioma cells by RapaLink-1, a third-generation mTOR kinase inhibitor [9]. Despite preceding research suggesting the anticancer effect of *FKBP1A*, the role of *FKBP1A* in glioblastoma and the underlying biologic mechanism remain unclear.

With the development of high-throughput sequencing technologies, we can obtain the gene expression profile of thousands of genes [10,11]. In the present study, we combined RNA-seq data and experiments to explore the detailed molecular mechanisms of *FKBP1A* involved in GBM, which will help us better understand GBM and offer a new potential treatment option.

## 2. Results

### 2.1. FKBP1A Was the Hub Gene in GBM Progression

**Figure 1:**
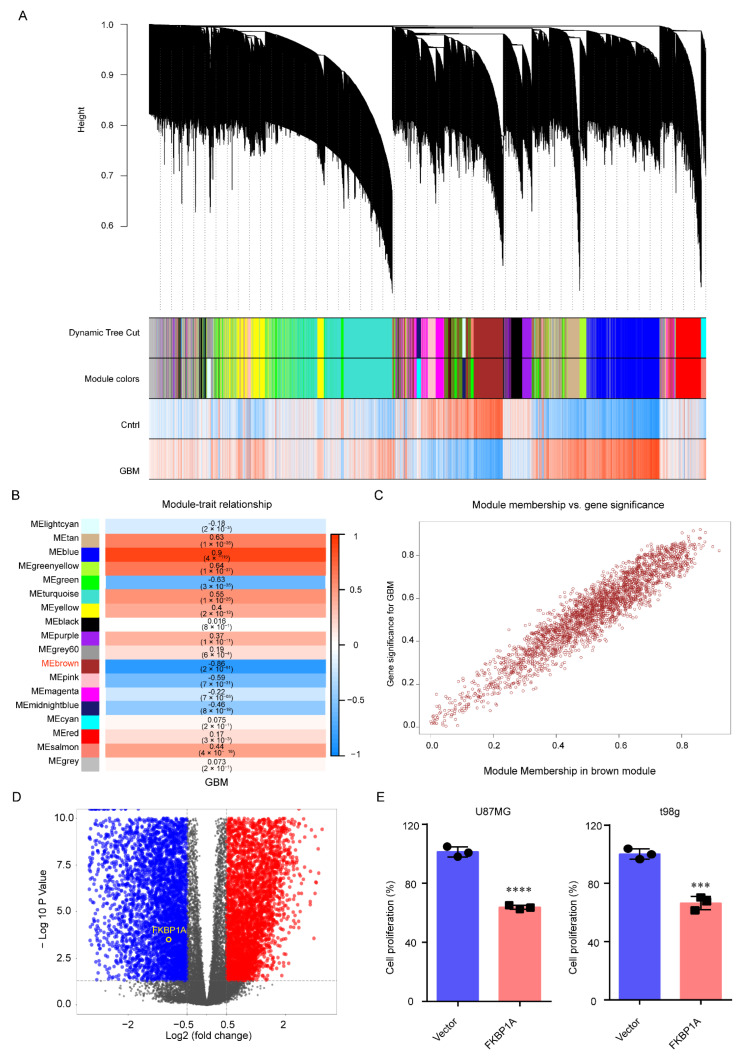
*FKBP1A* had a pivotal role in GBM cell growth. (**A**) Cluster dendrogram of 25,221 genes from 168 GBM and 142 normal tissues. (**B**) Heatmap of the correlation between module eigengenes and clinical traits (GBM). The correlation coefficient and *P* value are shown in each box. (**C**) The relationship between module membership and gene significance was visualized by scatter plot. The module membership and gene significance of *FKBP1A* were 0.61 and 0.71, respectively. (**D**) The volcano map of DEGs. The blue pots indicate downregulated DEGs and the red pots represent the upregulated DEGs. The cutoff of |Log_2_ fold change| > 0.5 and *p* < 0.05. (**E**) The proliferation of U87MG and t98g cells overexpressing *FKBP1A* for 48 h. The data are presented as the mean ± SD values. *** *p* < 0.001, **** *p* < 0.0001.

We identified *FKBP1A* as the hub gene in GBM via weighted gene correlation network analysis (WGCNA) and differential expression gene (DEG) analysis and then verified the results by conducting experiments. We first performed WGCNA to screen gene sets related to GBM by bulk RNA-seq data from TCGA and GTEx (168 GBM and 142 normal tissues, Figure 1A, Table S1). Based on the cutoff of WGCNA, the gene set of the brown module was identified as GBM suppressor genes (Pearson correlation value = −0.86, *p* < 0.05; Figure 1B, Table S2). Notably, *FKBP1A* was classified into the brown module, which suggested that *FKBP1A* might be the hub gene in GBM progression. Additionally, we calculated the relationship between the module membership and gene significance of *FKBP1A* in the brown module to be 0.61 and 0.71, respectively (Figure 1C), which further suggested that *FKBP1A* played a key role in GBM. 

Subsequently, we identified the key role of *FKBP1A* in GBM by DEG analysis. The mRNA expression of *FKBP1A* was significantly downregulated in GBM compared with the Vector groups (|Log_2_ Fold Change| > 0.5 and *p* < 0.05) based on the bulk RNA-seq data from TCGA and GTEx (Figure 1D). Together, we suggested that *FKBP1A* played a critical role in GBM by WGCNA and DEG analysis based on bulk RNA-seq data from TCGA and GTEx. Finally, we experimentally verified the bioinformatics results that suggested that *FKBP1A* was the hub gene in GBM. We found that the overexpression of *FKBP1A* significantly suppressed cancer cell growth in the U87MG and t98g (Figure 1E) GBM cell lines at 48h with CCK-8 assays (*p* < 0.001).

### 2.2. Exploring the Pathways of FKBP1A Involved in GBM

**Figure 2:**
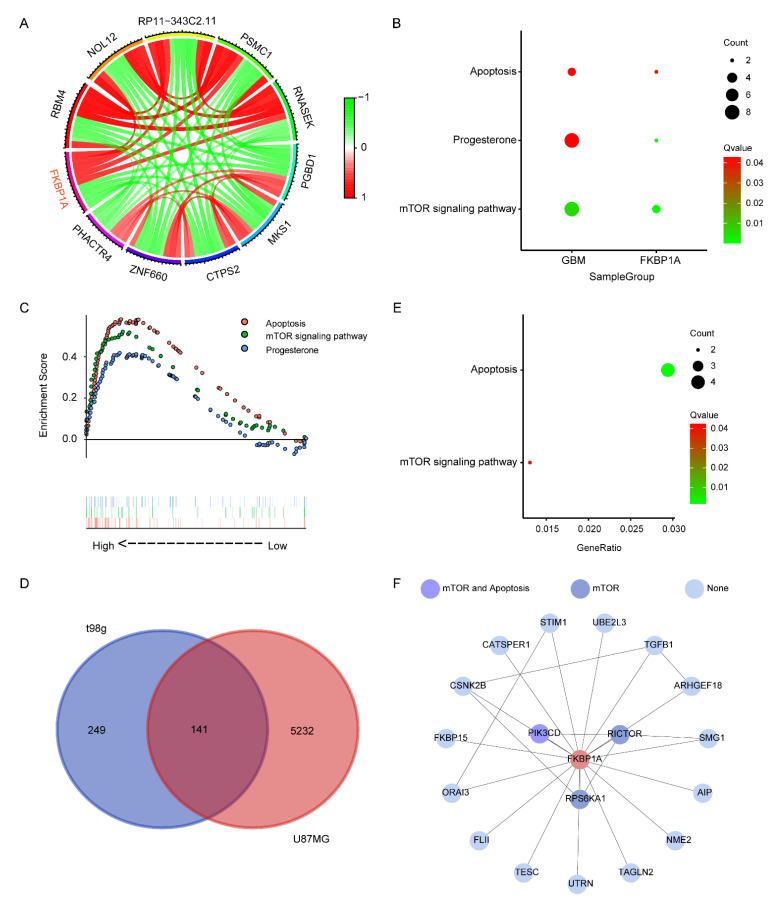
Identification of the *FKBP1A* signaling pathways involved in GBM. (**A**) The relationships between the top ten genes of *FKBP1A*. Red, positive association; green, negative association. The threshold of Pearson correlation value cutoff > 0.5 and *p* < 0.001. (**B**) KEGG over-representation test based on genes of the brown module of WGCNA and coexpressed genes of *FKBP1A*. The cutoff of significantly enriched pathways was *p* < 0.05. (**C**) The potential signaling pathways of *FKBP1A* in GBM were enriched between *FKBP1A* high- and low-expression groups by GSEA. The threshold was *p* < 0.05 and FDR < 0.05. (**D**) Venn plot of common DEGs based on the bulk RNA-seq data from the overexpression of *FKBP1A* in U87MG or t98g. The cutoff of |Log_2_ fold change| > 0.5 and *p* < 0.05. (**E**) KEGG over-representation test based on 141 common DEGs from the bulk RNA-seq data from the overexpression of *FKBP1A* in U87MG or t98g. The cutoff was |Log_2_ fold change| > 0.5 and *p* < 0.05. (**F**) The connections of *FKBP1A* and key pathways were constructed according to protein–protein interactions. Medium confidence > 0.4.

First, we explored the coexpression of *FKBP1A* in GBM based on the results of DEG analysis for the subsequent pathway enrichment analysis. A total of 2517 genes were identified to be coexpressed with *FKBP1A* based on the threshold of |correlation coefficient| > 0.5 and *p* < 0.001 (Table S3). The top 10 coexpressed genes (five positively coexpressed genes and five negatively coexpressed genes) with *FKBP1A* are shown in Figure 2A. We used two pathway enrichment methods to assess the potential signaling pathways involved in the antitumor effect of *FKBP1A* in GBM. We first performed the KEGG over-representation test based on the GBM-related genes screened by WGCNA to identify the pathways involved in GBM. A total of 47 KEGG terms were significantly enriched in GBM (*p* < 0.05, Table S4). Subsequently, we employed the KEGG over-representation test using the coexpression genes of *FKBP1A* to explore the potential pathways of *FKBP1A* in GBM. Thirty signaling pathways were significantly enriched (Table S5). Three common signaling pathways existed between the GBM and *FKBP1A* groups (Figure 2B). Simultaneously, we carried out a gene set enrichment analysis (GSEA) on the KEGG gene sets between *FKBP1A* high-expression and low-expression groups (Figure 2C, Table S6) to identify the critical pathways involved in *FKBP1A* in GBM. Three shared pathways enriched by both methods were shown, including ‘Apoptosis’, ‘Progesterone’, and ‘mTOR signaling pathway’. To further validate the above results, we also performed a pathway enrichment based on the bulk RNA-seq data from the overexpression of *FKBP1A* in U87MG or t98g. The individual heatmaps of the RNA-seq data of individually expressed genes were shown in Figure S1. Furthermore, 141 DEGs were shared between U87MG and t98g (Figure 2D, Table S7). Then, a pathway enrichment analysis based on the DEGs retrieved a total of 76 significant KEGG pathways (*p* < 0.05, Table S8). Two pathways were consistent with the above results from the TCGA GBM and GTEx cohort: ‘Apoptosis’ and the ‘mTOR signaling pathway’ (Figure 2E). Furthermore, three shared pathways were obtained by taking the intersection of the pathways of the KEGG over-representation test; GSEA between the *FKBP1A* high-expression and low-expression groups; and *FKBP1A* coexpressed genes, including ‘Apoptosis’, the ‘mTOR signaling pathway’, and ‘Pathways in cancer’ (Figure S2). Taken together, these analysis results strongly suggest that ‘Apoptosis’ and the ‘mTOR signaling pathway’ may play an important role in the suppression of GBM cell growth by *FKBP1A*. 

To visualize the relationship between *FKBP1A* and the cell signaling pathways, we constructed a protein–protein interaction (PPI) network with the genes of the WGCNA brown module and the *FKBP1A* coexpressed genes using the STRING dataset (combined score > 0.4). *FKBP1A* directly interacted with 18 genes (*PIK3CD*, *RICTOR*, *RPS6KA1*, *UBE2L3*, *TGFB1*, *ARHGEF18*, *SMG1*, *AIP*, *NME2*, *TAGLN2*, *UTRN*, *TESC*, *FLII*, *ORAI3*, *FKBP15*, *CSNK2B*, *CATSPER1*, and *STIM1*; Figure 2F) according to the shortest path analysis. *PIK3CD* was involved in both the mTOR signaling pathway and apoptosis pathway, while *RICTOR* and *RPS6KA1* were involved only with the mTOR signaling pathway. The other 16 genes had no association with the former enrichment pathways.

### 2.3. Verifying the Key Pathways in scRNA-seq of GBM

**Figure 3:**
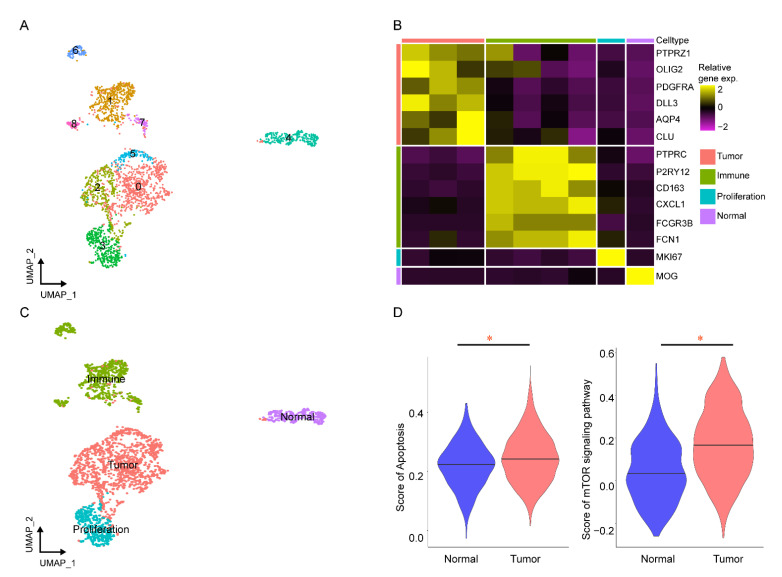
Verifying the key pathways of *FKBP1A* involved in GBM at the single-cell level. (**A**) The eight cell clusters were visualized by a UMAP plot. (**B**) Relative expression map of known marker genes associated with each cell type. (**C**) The UMAP plot of 2483 cells from twenty GBM cancers and seven corresponding precancerous tissue samples is color coded by the major cell types as indicated. (**D**) Comparison of apoptosis and mTOR signaling pathway gene set enrichment scores for the GSVA of single cells between tumors and normal cells, * *p* < 0.05.

After quality control with default parameters, 2483 cells remained for further analysis. Eight cell clusters were identified with a resolution of 0.2 and visualized by UMAP approaches (Figure 3A). Then, all cells were classified into four different types by canonical markers (Figure 3B). The four major cell types of GBM are shown in Figure 3C: tumor cells (*PTPRZ1*, *OLIG2*, *PDGFRA*, *DLL3*, *AQP4*, and *CLU*), immune cells (*PTPRC*, *P2RY12*, *CD163*, *CXCL1*, *FCGR3B*, and *FCN1*), proliferation cells (*MKI67*), and normal cells (*MOG*). Subsequently, we verified the key pathways (apoptosis and the mTOR signaling pathway) at a single-cell resolution with GSVA. We found that the two key pathways were all significantly enriched in tumor cells compared with normal cells (*P* < 0.05) (Figure 3D).

### 2.4. Experimentally Validating the Key Pathways

**Figure 4:**
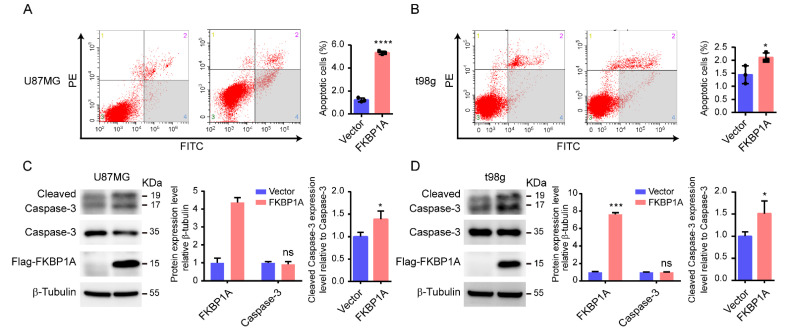
Role of *FKBP1A* in the apoptotic pathway of GBM cells. (**A**,**B**) U87MG (**A**) and t98g (**B**) cell apoptosis were analyzed by flow cytometry (left). The percentages of early apoptotic cells are shown on the right (n = 3). (**C**,**D**). Western blot analysis of U87MG (**C**) and t98g (**D**) cells transfected with Vector or *FKBP1A* plasmids. The data are presented as the mean ± SD values. * *p* < 0.05, *** *p* < 0.001, **** *p* < 0.0001. ns: no significant difference.

To investigate whether *FKBP1A* affects GBM through the apoptosis and mTOR signaling pathways, we examined the critical markers of the two pathways. By using a flow cytometry experiment, it was discovered that U87MG and t98g cells overexpressing *FKBP1A* had a much higher rate of cell apoptosis than the control group (Figure 4A,B). Further immunoblot results show that the overexpression of *FKBP1A* elevated the expression of cleaved-caspase 3, which was the key marker in the apoptotic pathway in both GBM cell lines (Figure 4C,D). These results confirm that *FKBP1A* regulated GBM via the apoptosis pathway. Unfortunately, the overexpression of *FKBP1A* could not affect the expression of the mammalian target of Rapamycin (mTOR), Akt1 (AKT Serine/Threonine Kinase 1), and Phosphatidylinositol 3-hydroxykinase (PI3K) proteins (Figure S3). It appeared that *FKBP1A* could not directly regulate the mTOR signaling pathway without exogenous stimuli such as Rapamycin. In particular, *FKBP1A* could significantly downregulate the epidermal growth factor receptor (EGFR), which was demonstrated in previous studies [12,13]. Generally, our results proved that upregulated *FKBP1A* killed the GBM cells by activating the apoptosis pathway.

## 3. Discussion

Glioblastoma (GBM) is an extremely aggressive brain tumor with a poor prognosis. We searched PubMed for clinical trial reports and reviews about GBM therapy published between 1 January 2009 and 1 December 2019. The search results showed that there is less than a 1.5 year median survival rate for GBM patients. Therefore, better therapeutic targets for GBM are urgently needed. 

In this study, by using WGCNA and DEGs, we identified *FKBP1A* as a hub gene in GBM. Furthermore, the CCK-8 assay verified that *FKBP1A* overexpression could inhibit GBM cell growth. Then, two signal pathway enrichment methods were used to identify the critical signal pathways involved in GBM by *FKBP1A*. The key signaling pathways were further verified by the scRNA-seq data of GBM and bulk RNA-seq data of *FKBP1A* overexpressing GBM cell lines. The apoptosis assay by flow cytometry and Western blot further demonstrated that GBM could participate in the progression of GBM through apoptosis signaling pathways.

*FKBP1A* belongs to the immunophilin family, which exerts various biological functions and is related to multiple tumors. Previous studies suggested that *FKBP1A* had both tumor-promoting and tumor-suppressive roles. In prostate cancer, by sequestering miR-515-5p, Circ-PAPPA enhanced malignant phenotypes of prostate cancer cells by enhancing the expression of *FKBP1A* [14]. The silencing of LncRNA AFAP1-AS1 could reduce *FKBP1A* expression through miR-195-5p, thereby improving paclitaxel tolerance in PTX-resistant prostate cancer cells [15]. Another LncRNA SNHG15 promoted prostate cancer progression by manipulating the miR-338-3p/*FKBP1A* axis [16]. On the contrary, Fong et al. found that *FKBP1A* could inhibit tumor invasion by increasing the anti-invasive Sdc1 gene and inhibiting the proinvasive MMP9 gene in mouse breast cancer and melanoma cells [6]. In addition, *FKBP1A* has been shown to enhance the sensitivity of tumor cells to chemotherapy. For instance, Liu et al. found that FKBP12 enhanced the sensitivity of cancer cells to chemotherapy and Nutlin-3 treatment by directly interacting with and degrading MDM2 [7]. In particular, a third-generation mTOR inhibitor, RapaLink-1, which inhibits mTORC1 and proliferation in human glioma cells, requires *FKBP1A* in order to be practical and durable [9]. This beneficial effect of *FKBP1A* on GBM treatment is consistent with the results of our study.

In this study, we determined that *FKBP1A* plays a crucial regulatory role in GBM. Firstly, we confirmed that *FKBP1A* is a hub gene of GBM by bioinformatics analysis. By WGCNA, we found that *FKBP1A* belonged to the gene set most significantly negatively associated with GBM. Subsequently, the differential analysis showed that *FKBP1A* was significantly downregulated in GBM (*p* < 0.05). Furthermore, we used the CCK-8 cell proliferation assay to verify that overexpressing *FKBP1A* could inhibit the cell growth of two GBM cell lines, U87MG and t98g. Our study proved that *FKBP1A* was involved in GBM progression by bioinformatics analysis and molecular biological techniques.

In this study, we further verified that *FKBP1A* manipulated the progression of GBM via the apoptosis pathway through bioinformatics and experiments. First, we determined the potential signaling pathways of *FKBP1A* participating in GBM through the pathway enrichment methods of the KEGG over-representation test and GSEA. Furthermore, bulk RNA-seq data from *FKBP1A* overexpressing cell lines revealed that *FKBP1A* might be involved in GBM through the apoptosis and mTOR signaling pathways. In addition, this study prospectively validated the two key signaling pathways at the single-cell level. Then, we confirmed that *FKBP1A* upregulated the expression level of cleaved caspase-3, which was a key executor of apoptosis, inducing apoptosis via caspase activation. Previous research showed that *FKBP1A* silencing in breast tumor cells weakened doxorubicin-induced cytotoxicity. Conversely, overexpressing *FKBP1A* exhibited a more potent inhibition of MDM2 by doxorubicin, resulting in stronger cytotoxic and apoptotic effects [8], which corroborated our results. 

Bioinformatics analysis confirmed that *FKBP1A* downregulated the mTOR signaling pathway at the mRNA level. However, when *FKBP1A* was overexpressed without other stimulants such as Rapamycin, no changes in the expression of the mTOR signaling pathway proteins were detected except for EGFR (Figure S3). We speculated that changes in the expression of other signals might have compensated for the disorder of the mTOR signaling pathways during the overexpression of *FKBP1A*. As for EGFR, it has been reported that *FKBP1A* inhibits the autophosphorylation of the EGFR by interacting with it [12,13].

## 4. Materials and Methods

### 4.1. Experiment Design

**Figure 5:**
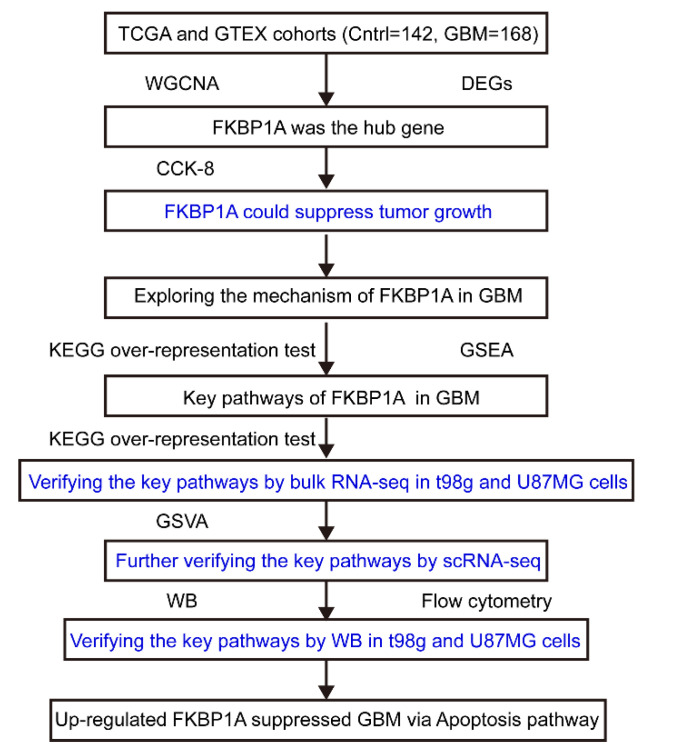
The workflow of the present study. GBM: glioblastoma multiforme, WGCNA: weighted correlation network analysis, DEG: differentially expressed gene, KEGG: Kyoto Encyclopedia of Genes and Genomes, GSEA: gene set enrichment analysis, GSVA: gene set variation analysis, CCK-8: cell counting kit-8, WB: Western blot.

The workflow of the present study is shown in Figure 5. Briefly, we first proved that *FKBP1A* was the hub gene in GBM with a weighted gene coexpression network analysis (WGCNA) and differential expression analysis. Following that, the results of the bioinformatics analysis were validated with a CCK-8 assay, and we found that the overexpression of *FKBP1A* could repress the growth of the U87MG and t98g GBM cells. Secondly, we explored the signaling pathway of *FKBP1A* involved in GBM by the KEGG over-representation test and gene set enrichment analysis (GSEA). Moreover, we verified the key pathways with a bulk RNA-seq of the *FKBP1A*-overexpressed U87MG and t98g GBM cell lines. Additionally, we evaluated the key pathway in the scRNA-seq of GBM at the single cell level. We further performed Western blots and flow cytometry on U87MG and t98g GBM cell lines to identify key pathways.

### 4.2. Identification of Gene Sets Related to GBM 

We aimed to screen the antitumor gene sets, which could promote tumor regression when upregulated in the tumor. We employed WGCNA to obtain the genes related to GBM by WGCNA v 1.70 R packages [17]. Briefly, we first selected 25,221 genes from 310 samples to construct the coexpression network for WGCNA. Subsequently, we defined the adjacency matrix with the soft thresholding power value (power = 6). Finally, we used the dynamic tree cut algorithm to obtain the modules of gene expression with the parameters (minModulesize = 50, mergeCutHeight = 0.15, and deepSplit = 1). Genes in the WGCNA module with the lowest correction value and *p* < 0.05 significantly selected the GBM suppressor gene sets. Highly connected intramodular hub genes tend to have high module membership values to the respective module. Higher gene significance represents a higher correlation between the gene and the target traits. Therefore, genes with a cor. Gene module membership > 0.5 and cor. Gene significance > 0.5 were defined as hub genes [18].

### 4.3. Differential Expression Genes between GBM and Normal Groups

The bulk RNA-seq data (count value) from GTEx and TCGA GBM were obtained from UCSC XENA (http://xena.ucsc.edu/ accessed on 15 June 2020). The GBM cohort consisted of 142 GBM patient tumor tissues and 168 normal tissues samples. Then, we employed a differential expression analysis based on the GBM datasets with the cutoff of |Log_2_ fold change| > 0.5 and *p* < 0.05 by the limma v 3.46.0 R packages [19]. We further performed an analysis of the coexpression of *FKBP1A* on the differentially expressed genes (DEGs) using the threshold of the Pearson correlation value cutoff > 0.5 and *p* < 0.001.

### 4.4. Identification of FKBP1A Signaling Pathways Involved in GBM

We combined two methods to screen the *FKBP1A* signaling pathways involved in GBM. First, using WGCNA, we identified the pathways enriched in GBM by the KEGG over-representation test based on the GBM-related gene sets. Based on the genes of the brown module of WGCNA and the coexpression genes of *FKBP1A* (Pearson value > 0.5 and *p* < 0.001), we conducted the KEGG over-representation test [20]. The cutoff of significantly enriched pathways was *p* < 0.05. Simultaneously, the putative signaling pathways of *FKBP1A* in GBM between high and low *FKBP1A* expression groups were examined using GSEA v 4.0.3. with the thresholds *p* < 0.05 and FDR < 0.05 [21]. Finally, we defined the overlapping signaling pathways in the two different algorithms as the key pathways of *FKBP1A* involved in GBM.

### 4.5. ScRNA-Seq Data Collection

To further verify the key pathways of *FKBP1A* involved in GBM, we downloaded the scRNA-seq of GBM from the Gene Expression Omnibus (GEO, GSE117891, 10 June 2021) [22]. After quality assessment, we selected twenty GBM cancers and seven corresponding precancerous tissue samples for subsequent analyses. Then, we employed Seurat v 4.02 [23] to identify the major cell types based on the canonical markers. Gene set variation analysis (GSVA) calculates sample-wise gene set enrichment scores as a function of the genes inside and outside the gene set, analogously to a competitive gene set test. We performed GSVA to verify the key pathways of *FKBP1A* involved in GBM using the GSVA v 1.38.2 R package [24]. 

### 4.6. Potential Connections between FKBP1A and Critical Pathways

We constructed the protein–protein network using coexpression genes of *FKBP1A* and the brown gene set screened by WGCNA based on the STRING database with medium confidence > 0.4 [25]. Then, we used a shortest-pathway analysis to identify the potential connections between *FKBP1A* and key pathways with the NetworkX v 2.5 Python package (https://github.com/networkx/networkx (accessed on 19 June 2022)). 

### 4.7. Cell Culture and Plasmids Transfection

The U87MG human glioblastoma cell line was purchased from the Chinese Academy of Sciences Cell Bank (Shanghai, China). The t98g human glioblastoma cell line was kindly provided by the School of Pharmaceutical Sciences, Sun Yat-sen University (Guangzhou, China). All of these cells were cultivated in Dulbecco’s modified Eagle’s medium from Gibco (Waltham, MA, USA), which was supplemented with a 10% fetal bovine serum and 1% penicillin-streptomycin. They were all kept at 37 °C in a 5% CO_2_ humidified environment. Flag-*FKBP1A* and correspondent Vector plasmids were purchased from WZ Biosciences Inc (Jinan, China). As for the transient transfection of Flag-*FKBP1A* or Vector plasmids, U87MG or t98g cells were seeded in 6-well plates and grown to 70% confluence. Plasmid transfection was performed with the Lipofectamine™ 3000 Transfection Reagent (Invitrogen, Carlsbad, CA, USA) according to the manufacturer’s instructions. Extracts of the proteins were obtained 48 h after the plasmids were transfected.

### 4.8. Cell Proliferation Assay

The cell counting kit-8 reagent was applied to detect the cell proliferation experiment (CCK-8, Dojindo, Kumamoto, Japan). Protocols provided by the manufacturer were followed during the assays. U87MG and t98g cells were transfected with Flag-*FKBP1A* or Vector plasmids for 48 h before detection.

### 4.9. RNA Isolation, Sequencing, and Analysis

*FKBP1A*-overexpressed U87MG and t98g GBM cells were gathered and aggregated, and each treatment was carried out in triplicate for the RNA-Seq analysis. The total RNA was isolated from the cell lines in the Vector and Flag-*FKBP1A* groups using a TRIzol reagent (Invitrogen, Carlsbad, CA, USA). Following a standardized process, libraries were built using high-quality RNA samples (28 S/18 S = 2.0–2.2, RIN > 9.0) on the Illumina NovaseqTM 6000 platform (Lianchuan Biotechnology Co. Ltd., Hangzhou, China) using the double terminal sequencing method. After sequencing, reads of poor quality, adaptor contamination, and a high concentration of unknown bases (N) were removed to produce clean reads by FastQC v 0.11.9 [26], and clean reads were then mapped to the Homo sapiens reference genome (GRCh38) by HISAT v 2.2.0 [27]. Then, we performed a differential expression analysis with the cutoff of |Log_2_ fold change| > 0.5 and *p* < 0.05 by DESeq2 v 1.30.1 R packages between *FKBP1A*-overexpressed U87MG cells and control U87MG cells (*FKBP1A*-overexpressed t98g cells and control t98g cells). Subsequently, we employed KEGG over-representation test pathway enrichment to evaluate the key pathways of *FKBP1A* involved in GBM with the cutoff *p* < 0.05.

### 4.10. Cell Apoptosis Analysis

Cell apoptosis was detected using a FITC Annexin V Apoptosis Detection Kit (BD, Franklin Lakes, NJ, USA). Briefly, U87MG or t98g cells were transfected with Flag-*FKBP1A* or Vector plasmids for 48 h. Then, the cells were washed three times with cold PBS and collected in 100 μL of a 1× binding buffer. A total of 5 μL of FITC Annexin V and 5 μL of PI were added to the cell suspension at room temperature for 15 min. Next, in each sample, 450 μL of the binding buffer were added and analyzed by flow cytometry. 

### 4.11. Western Blot

A Western blot was performed as previously described [28]. The following primary antibodies were used: human cleaved caspase-3, human caspase-3, human *FKBP1A* antibody, and human α-actinin antibody purchased from Cell Signaling Technology (Danvers, MA, USA); the human β-Tubulin antibody was obtained from Sigma-Aldrich (St. Louis, MO, USA).

### 4.12. Statistical Analysis

All data are presented as mean ± SD, and a Student’s *t* test was used to assess the differences between the two groups with R v 4.0.5. *p* < 0.05 was considered statistically significant.

## 5. Conclusions

In conclusion, the present study demonstrated that *FKBP1A* could regulate GBM progression through the apoptosis pathway by integrating a bioinformatics analysis with experiments, which provided a better understanding of GBM and a new potential for treating it.

## Data Availability

Raw sequencing data are available in NCBI’s BioProject SRA database (PRJNA897117).

## Supplementary Materials

The following supporting information can be downloaded at: https://www.mdpi.com/article/10.3390/ijms232314935/s1, Figure S1: Heatmap of differentially expressed genes of RNA-seq; Figure S2: Venn plot of enriched pathways of KEGG over-representation test based on 141 common DEGs, GSEA between *FKBP1A* high-expression and low-expression groups, and *FKBP1A* co-expressed genes; Figure S3: Western blot of *FKBP1A* overexpression in GBM cell lines; Table S1: Detailed clinical data and grouping information from TCGA and GTEX cohorts; Table S2: The genes identified by WGCNA in the brown module; Table S3: The co-expression genes of *FKBP1A*; Table S4: The enriched pathways based on genes screened by WGCNA; Table S5: The enriched pathways based on the co-expression genes of *FKBP1A*; Table S6: The GSEA results in *FKBP1A* high expression groups; Table S7: The 141 Differential expression genes shared between U87MG and t98g; Table S8: The enriched pathways based on the 141 common differential expression genes between U87MG and t98g.

## References

[1] McKinnon C., Nandhabalan M., Murray S.A., Plaha P. (2021). Glioblastoma: Clinical presentation, diagnosis, and management. BMJ.

[2] Tan A.C., Ashley D.M., Lopez G.Y., Malinzak M., Friedman H.S., Khasraw M. (2020). Management of glioblastoma: State of the art and future directions. CA Cancer J. Clin..

[3] Brodbelt A., Greenberg D., Winters T., Williams M., Vernon S., Collins V.P., National Cancer Information Network Brain Tumour Group (2015). Glioblastoma in England: 2007-2011. Eur. J. Cancer.

[4] Stupp R., Mason W.P., van den Bent M.J., Weller M., Fisher B., Taphoorn M.J., Belanger K., Brandes A.A., Marosi C., Bogdahn U. (2005). Radiotherapy plus concomitant and adjuvant temozolomide for glioblastoma. N. Engl. J. Med..

[5] Tong M., Jiang Y. (2015). FK506-Binding Proteins and Their Diverse Functions. Curr. Mol. Pharmacol..

[6] Fong S., Mounkes L., Liu Y., Maibaum M., Alonzo E., Desprez P.Y., Thor A.D., Kashani-Sabet M., Debs R.J. (2003). Functional identification of distinct sets of antitumor activities mediated by the FKBP gene family. Proc. Natl. Acad. Sci. USA.

[7] Liu T., Xiong J., Yi S., Zhang H., Zhou S., Gu L., Zhou M. (2017). FKBP12 enhances sensitivity to chemotherapy-induced cancer cell apoptosis by inhibiting MDM2. Oncogene.

[8] Xing M., Wang J., Yang Q., Wang Y., Li J., Xiong J., Zhou S. (2019). FKBP12 is a predictive biomarker for efficacy of anthracycline-based chemotherapy in breast cancer. Cancer Chemother. Pharmacol..

[9] Fan Q., Aksoy O., Wong R.A., Ilkhanizadeh S., Novotny C.J., Gustafson W.C., Truong A.Y., Cayanan G., Simonds E.F., Haas-Kogan D. (2017). A Kinase Inhibitor Targeted to mTORC1 Drives Regression in Glioblastoma. Cancer Cell.

[10] Stark R., Grzelak M., Hadfield J. (2019). RNA sequencing: The teenage years. Nat. Rev. Genet..

[11] Hwang B., Lee J.H., Bang D. (2018). Single-cell RNA sequencing technologies and bioinformatics pipelines. Exp. Mol. Med..

[12] Mathea S., Li S., Schierhorn A., Jahreis G., Schiene-Fischer C. (2011). Suppression of EGFR autophosphorylation by FKBP12. Biochemistry.

[13] Lopez-Ilasaca M., Schiene C., Kullertz G., Tradler T., Fischer G., Wetzker R. (1998). Effects of FK506-binding protein 12 and FK506 on autophosphorylation of epidermal growth factor receptor. J. Biol. Chem..

[14] Wang G., Zhao H., Duan X., Ren Z. (2021). CircRNA pappalysin 1 facilitates prostate cancer development through miR-515-5p/*FKBP1A* axis. Andrologia.

[15] Leng W., Liu Q., Zhang S., Sun D., Guo Y. (2020). LncRNA AFAP1-AS1 modulates the sensitivity of paclitaxel-resistant prostate cancer cells to paclitaxel via miR-195-5p/*FKBP1A* axis. Cancer Biol. Ther..

[16] Zhang Y., Zhang D., Lv J., Wang S., Zhang Q. (2019). LncRNA SNHG15 acts as an oncogene in prostate cancer by regulating miR-338-3p/*FKBP1A* axis. Gene.

[17] Langfelder P., Horvath S. (2008). WGCNA: An R package for weighted correlation network analysis. BMC Bioinform..

[18] Xie Z., Li X., He Y., Wu S., Wang S., Sun J., He Y., Lun Y., Zhang J. (2020). Immune Cell Confrontation in the Papillary Thyroid Carcinoma Microenvironment. Front. Endocrinol..

[19] Ritchie M.E., Phipson B., Wu D., Hu Y., Law C.W., Shi W., Smyth G.K. (2015). limma powers differential expression analyses for RNA-sequencing and microarray studies. Nucleic Acids Res..

[20] Kanehisa M., Goto S. (2000). KEGG: Kyoto encyclopedia of genes and genomes. Nucleic Acids Res..

[21] Subramanian A., Tamayo P., Mootha V.K., Mukherjee S., Ebert B.L., Gillette M.A., Paulovich A., Pomeroy S.L., Golub T.R., Lander E.S. (2005). Gene set enrichment analysis: A knowledge-based approach for interpreting genome-wide expression profiles. Proc. Natl. Acad. Sci. USA.

[22] Yu K., Hu Y., Wu F., Guo Q., Qian Z., Hu W., Chen J., Wang K., Fan X., Wu X. (2020). Surveying brain tumor heterogeneity by single-cell RNA-sequencing of multi-sector biopsies. Natl. Sci. Rev..

[23] Hao Y., Hao S., Andersen-Nissen E., Mauck W.M., Zheng S., Butler A., Lee M.J., Wilk A.J., Darby C., Zager M. (2021). Integrated analysis of multimodal single-cell data. Cell.

[24] Hanzelmann S., Castelo R., Guinney J. (2013). GSVA: Gene set variation analysis for microarray and RNA-seq data. BMC Bioinform..

[25] von Mering C., Jensen L.J., Snel B., Hooper S.D., Krupp M., Foglierini M., Jouffre N., Huynen M.A., Bork P. (2005). STRING: Known and predicted protein-protein associations, integrated and transferred across organisms. Nucleic Acids Res..

[26] Rostovskaya M., Andrews S., Reik W., Rugg-Gunn P.J. (2022). Amniogenesis occurs in two independent waves in primates. Cell Stem Cell.

[27] Zhang Y., Park C., Bennett C., Thornton M., Kim D. (2021). Rapid and accurate alignment of nucleotide conversion sequencing reads with HISAT-3N. Genome Res..

[28] Xiang Y., Wang Q., Guo Y., Ge H., Fu Y., Wang X., Tao L. (2019). Cx32 exerts anti-apoptotic and pro-tumor effects via the epidermal growth factor receptor pathway in hepatocellular carcinoma. J. Exp. Clin. Cancer Res..

