# Supplementary material for: Upregulated FKBP1A Suppresses Glioblastoma Cell Growth via Apoptosis Pathway
Source: Int J Mol Sci. 2022 Nov 29;23(23):14935. doi: 10.3390/ijms232314935 (PMC9739687; doi:10.3390/ijms232314935)
Supplement: Supplementary file 1 [file ijms-23-14935-s001.zip › Supplementary meterials/ijms-1989999-supplementary.docx]

Upregulated FKBP1A Suppresses Glioblastoma Cell Growth via Apoptosis Pathway

Shaoyi Cai^1#^, Zhiyou Chen^1#^, Heng Tang^1^, Siyan Meng^1^, Liang Tao^1*^, Qin Wang^1^^*^

# These authors contributed equally to this work.

* Corresponding author. Email: wangqin6@mail.sysu.edu.cn; [taol@mail.sysu.edu.cn](mailto:taol@mail.sysu.edu.cn)

**Affiliations**

1. Department of Pharmacology, Zhongshan School of Medicine, Sun Yat-Sen University, Guangzhou 510080, China.


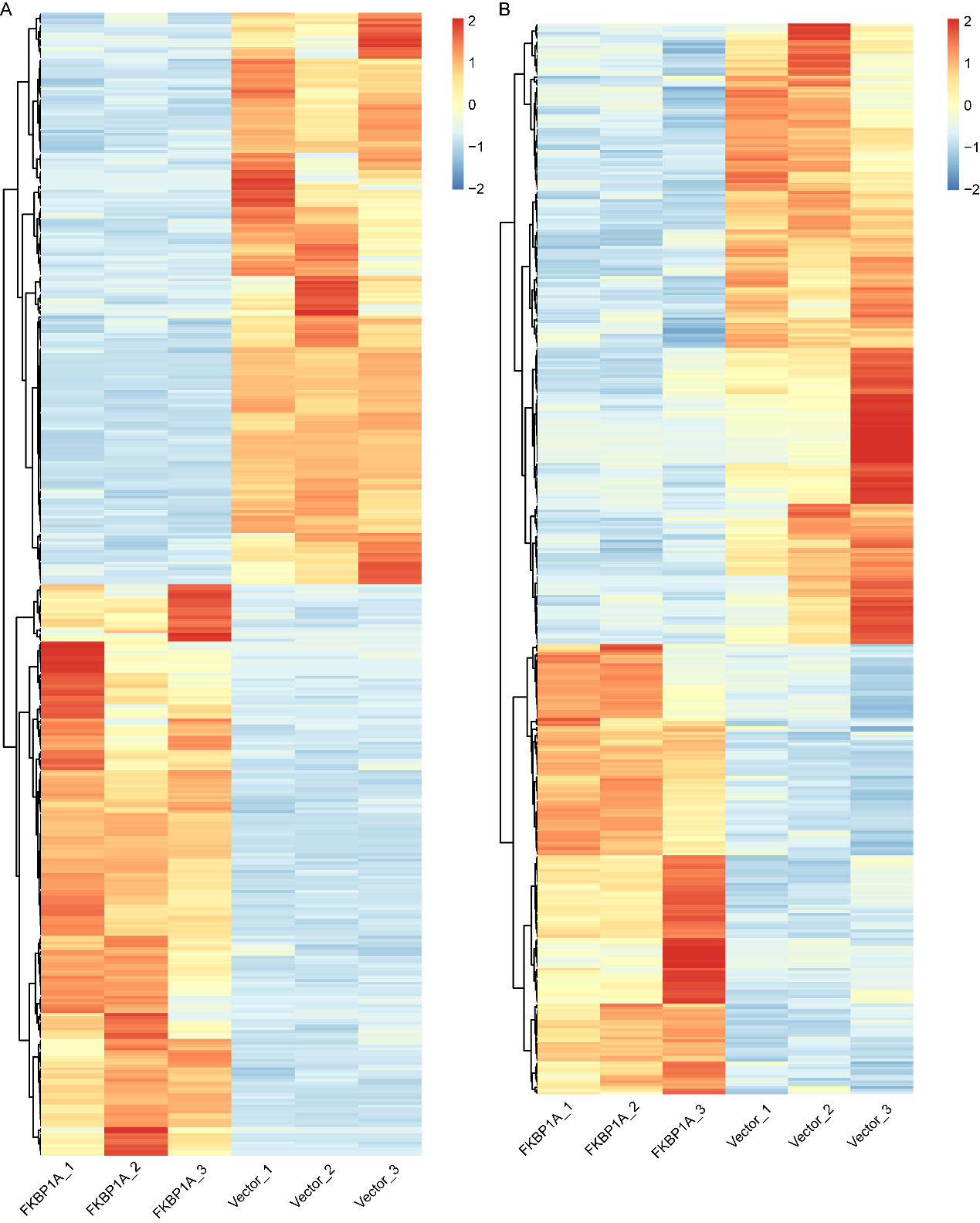
**Figure S1**. Heatmap of differentially expressed genes of RNA-seq. (A) The heatmap shows the top 200 up-regulated and the top 200 down-regulated differential genes between the FKBP1A and Vector groups in U87MG cells. (B) The heatmap shows the differential genes between the FKBP1A and Vector groups in t98g cells. The cutoff of |Log_2_ fold change| > 0.5 and *P* < 0.05.


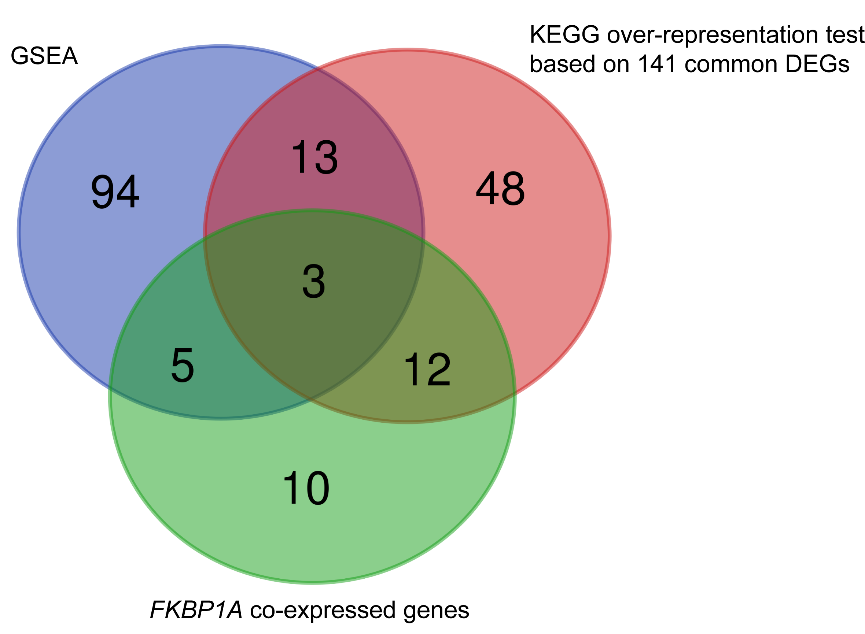


**Figure S2.** Venn plot of enriched pathways of KEGG over-representation test based on 141 common DEGs, GSEA between *FKBP1A* high-expression and low-expression groups, and *FKBP1A* co-expressed genes.


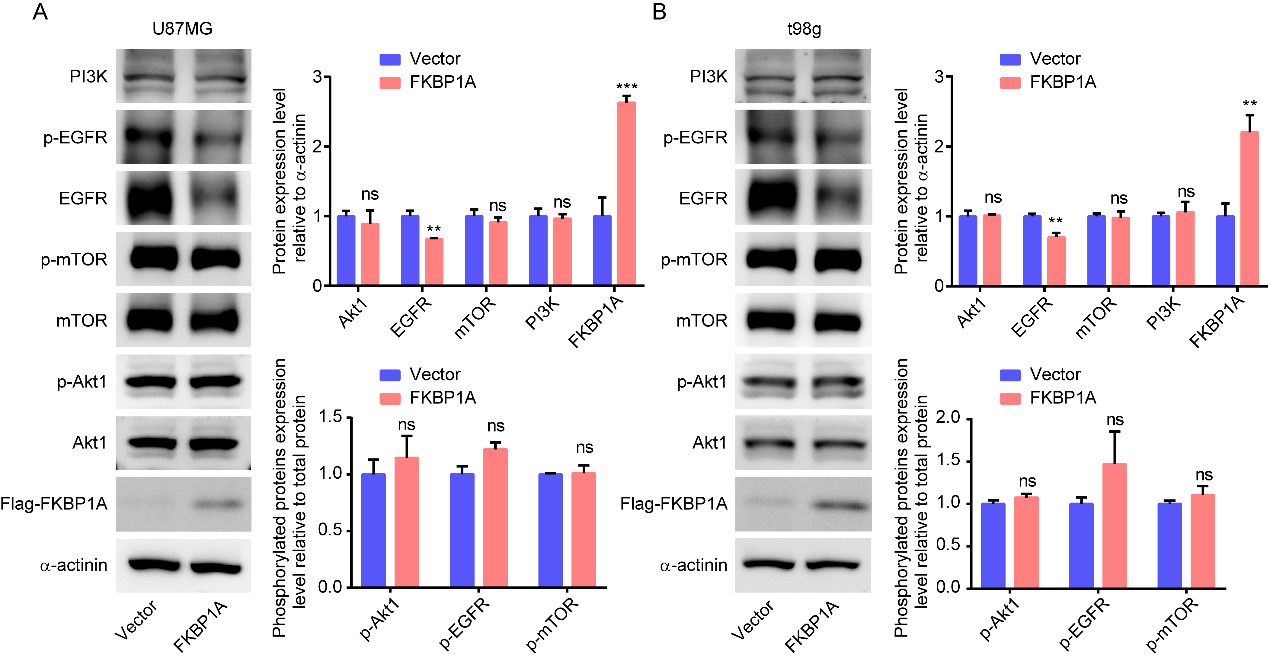


**Figure S3.** Western blot of FKBP1A overexpression in GBM cell lines.

**(A-B)** Flag-FKBP1A or Vector plasmids were transfected into U87MG **(A)** and t98g **(B)** cells for 48h. Proteins of mTOR signaling pathway were detected (left) and the relative protein quantification were shown on the right.
